# Genome-wide identification, evolution, and expression analysis of the NAC gene family in chestnut (*Castanea mollissima*)

**DOI:** 10.3389/fgene.2024.1337578

**Published:** 2024-01-25

**Authors:** Fei Cao, Chunlei Guo, Xiangyu Wang, Xuan Wang, Liyang Yu, Haie Zhang, Jingzheng Zhang

**Affiliations:** ^1^ College of Horticulture Science and Technology, Hebei Normal University of Science and Technology, Qinhuangdao, Hebei, China; ^2^ Engineering Research Center of Chestnut Industry Technology, Ministry of Education, Hebei Normal University of Science and Technology, Qinhuangdao, Hebei, China; ^3^ The Office of Scientific Research, Hebei Normal University of Science and Technology, Qinhuangdao, Hebei, China; ^4^ Hebei Collaborative Innovation Center of Chestnut Industry, Qinhuangdao, Hebei, China

**Keywords:** Castanea mollissima, NAC TF, duplication model, evolution, expression patterns

## Abstract

The NAC gene family is one of the most important transcription factor families specific to plants, responsible for regulating many biological processes, including development, stress response, and signal transduction. However, it has not yet been characterized in chestnut, an important nut tree species. Here, we identified 115 *CmNAC* genes in the chestnut genome, which were divided into 16 subgroups based on the phylogenetic analysis. Numerous *cis*-acting elements related to auxin, gibberellin, and abscisic acid were identified in the promoter region of *CmNACs*, suggesting that they play an important role in the growth and development of chestnut. The results of the collinear analysis indicated that dispersed duplication and whole-genome-duplication were the main drivers of *CmNAC* gene expansion. RNA-seq data of developmental stages of chestnut nut, bud, and ovule revealed the expression patterns of *CmNAC* genes. Additionally, qRT-PCR experiments were used to verify the expression levels of some *CmNAC* genes. The comprehensive analysis of the above results revealed that some *CmNAC* members may be related to chestnut bud and nut development, as well as ovule fertility. The systematic analysis of this study will help to increase understanding of the potential functions of the *CmNAC* genes in chestnut growth and development.

## Introduction

Transcription factors (TFs) are a type of specific protein molecules that regulate the expression intensity of specific genes by specifically binding to *cis*-acting elements, thereby affecting numerous life activities (Singh et al., 2002). NAC is a family of transcription factors unique to plants, named after the initial letters of NAM (no apical meristem), ATAF (Arabidopsis transcription activation factor), and CUC (cup-shaped cotyledon) (Nakashima et al., 2012). The NAC proteins can be divided into N-terminal conserved DNA binding regions and C-terminal variable transcriptional regulatory regions (Ooka et al., 2004). The structure domain of NAC is mainly located in the DNA binding region, consisting of approximately 150 amino acid residues, which further form five functionally diverse subdomains based on conservation (Jensen et al., 2010; Puranik et al., 2012). At present, the NAC TF has been characterized in numerous species, such as *Arabidopsis thaliana* (Lindemose et al., 2014), *Oryza sativa* (Nuruzzaman et al., 2010), *Glycine* max (Singh et al., 2021), *Capsicum annuum* (Diao et al., 2018), *Brassica rapa* (Liu et al., 2014), *Zanthoxylum bungeanum* (Hu et al., 2022), and *Camellia sinensis* (Wang et al., 2016).

Previous studies have found that NAC TFs are involved in many aspects of plant development, such as cell division, seed germination, leaf senescence, fruit maturation, and stress response (Xie et al., 2001; He et al., 2005; Kim et al., 2006). For example, *ANTHER INDEHISCENCE FACTOR* (*AIF*) in *Arabidopsis thaliana* (*A. thaliana*) is ectopically expressed during flowering development, resulting in another dehiscence and sterile phenotype (Shih et al., 2014). The NAC transcription factor *GhFSN5* in cotton (*Gossypium spp*) is heterologously expressed in *A*. *thaliana*, resulting in smaller pods and severe infertility (Sun et al., 2020). Overexpression of one NAC TF, *TaRNAC1*, mainly expressed in the roots of wheat (*Triticum aestivum*) can improve its root length, biomass, and drought resistance (Chen et al., 2018). Overexpression of *OsNAC6* and *OsSNAC2* in rice (*Oryza sativa*) can improve drought resistance of seedlings, salt, and cold stress (Hu et al., 2008). In tomatoes (*Solanum lycopersicum*), the NAC transcription factor gene *SlNAM1* binds to the promoters of two key genes for ethylene synthesis, *SlACS2* and *SlACS4*, and activates their expression, promoting ethylene synthesis, while the *SlNAM1* mutant delays tomato maturation (Gao et al., 2021). Overall, the functions of the NAC TFs in plants are diverse, and it is worth conducting in-depth research to provide potential value for molecular breeding of related species.

Chestnuts (*Castanea mollissima*) hold significant value as a food ingredient, with their rich nutritional profile and versatility. Chestnuts are packed with carbohydrates, proteins, and fats, making them an important source of essential nutrients for the human body (Massantini et al., 2021; Li et al., 2022). They are also a good source of vitamin C and B-complex vitamins, which boost immunity, promote metabolism, and provide energy (Barros et al., 2011; Li et al., 2022). Furthermore, chestnuts are a great source of dietary fiber, which aids in promoting digestive health, preventing constipation, and reducing cholesterol levels. Dietary fiber also helps control blood sugar levels, making it an important component of a diabetic-friendly diet. In addition, chestnuts have medicinal properties and are often used as herbal remedies. They contain abundant antioxidants that help prevent chronic diseases such as cardiovascular issues and cancer. Chestnuts also possess analgesic and anti-inflammatory properties, making them useful in relieving arthritis and other pain-related conditions (Mani et al., 2021; Nam et al., 2022). To conclude, chestnuts hold significant value as a food ingredient. They provide a rich nutritional profile and play an important role in healthcare. The versatility and health benefits appeal make chestnut a highly valuable woody plant for development.

In this study, chestnut genome data and bioinformatics analysis methods were fully utilized to understand the basic information, phylogenetic evolution, gene structure, motif composition, duplication patterns, and *cis*-acting elements of NAC gene family members in chestnut. In addition, RNA-seq data analysis was conducted on developmental stages of chestnut nut, bud and ovules, to understand the expression profiles of *CmNACs* during chestnut development. qRT-PCR and subcellular location experiments were used to validate the expression profile of the *CmNAC* genes. These works provided a reference for studying the functions of *NAC* genes in chestnuts.

## Materials and methods

### Identification and physicochemical properties

The genome data and annotation files of chestnut (N11-1) were downloaded from the Castanea Genome Database (http://castaneadb.net/), and the published NAC gene family members in *A. thaliana* were downloaded from TAIR (https://www.arabidopsis.org/) (Jensen et al., 2010). The Hidden Markov Model (HMM) file of the NAC domain (PF02365) was obtained from the Pfam database (http://pfam-legacy.xfam.org/). Firstly, the protein sequences of the *NAC* genes in *A*. *thaliana* were used as reference sequences to perform a Basic Local Alignment Search Tool (BLAST) program on all protein sequences of chestnut with the following parameters: evalue 1e-5 -outfmt 6. Subsequently, the HMM file of the NAM domain was used to search against the proteins of chestnut using HMMER 3.0 with an E-value of 1e-5. Then, we used Batch-CDD to confirm that the NAM domains existed in the candidate genes. Finally, 115 *NAC* genes were identified in the chestnut genome. ExPasy website (http://web.expasy.org/protparam/) was used to calculate the physicochemical properties of NAC proteins in chestnut.

### Phylogenetic and sequence analysis

A total of 209 NAC protein sequences from chestnut (115) and *A*. *thaliana* (94) (Jensen et al., 2010) were merged for phylogenetic analysis. MEGA 7.0 (Kumar et al., 2016) was used to construct the phylogenetic tree of *NAC* genes with the maximum likelihood. The “Find Best DNA/Protein Models (ML)” function in MEGA7.0 was used to find the best amino acid substitution model (partial deletion 95%), and the final parameters were as follows: Jones–Taylor–Thornton (JTT) model; Gamma Distributed (G); Partial deletion 95%; 1,000 bootstrap replications. Additionally, the neighbor-joining method was also used to construct a phylogenetic tree with the following parameters: Poisson model, pairwise deletion, and 1,000 bootstrap replications. MEME websites (http://meme.nbcr.net/meme/intro.html) were used to predict the conserved motifs of CmNAC proteins, with the following parameters: the number of repetitions, zero or one; and the maximum number of motifs, 10. The CmNAC proteins were submitted to NCBI-CDD Search to predict their conserved domains with default parameters. TBtools (Chen et al., 2020) was used to visualize the gene structure, conserved domains, and motifs of CmNAC members. We also constructed phylogenetic trees containing only members of the CmNAC gene family with methods the maximum likelihood method and neighbor-joining method, using the same strategy and method.

### Collinear and *cis*-acting elements analysis

The position information and gene density file of the target sequence were extracted using TBtools software, and visualized using the Gene Location program. The Multiple Collinearity Scan toolkit (MCScanX) software was used to conduct collinear analysis of the genomes of chestnut and *A. thaliana*, rice, oak (*Quercus robur*), and grape (*Vitis vinifera*) to explore the evolution of *CmNAC* genes, with default parameters (Wang et al., 2012). Notably, we identified the *CmNAC* genes formed by whole-genome-duplication (WGD) events, as we did before (Yu et al., 2023b). Specifically, we drew the homologous collinear gene dot-plot within the chestnut genome. The “add_ka-and_ks_to collinearity” in MCScanX was used to obtain the non-synonymous (Ka) and synonymous substitution sites (Ks) values of homologous gene pairs, and the median Ks values of collinear blocks were calculated by writing the script (Yu et al., 2023b). The collinear blocks in the homologous gene dot-plot were colored differently based on different median Ks values, which will help to locate the blocks formed by different duplication events. Finally, based on the distribution of Ks corresponding to the WGD event that occurred in the chestnut genome before (Yu et al., 2023b), combined with the complementarity of the collinear blocks, we identified the *CmNAC* genes formed by the WGD event. In addition, we also obtained *CmNAC* members from other duplication models, such as segmental, proximal, dispersed, and tandem duplication, from the result file of collinear analysis of the chestnut genome. The 2000 bp nucleic acid sequences upstream of the transcription initiation of 115 *CmNAC* genes were submitted to the PlantCARE website to predict the composition of *cis*-acting elements, and the results were classified into functional categories.

### Expression analysis of *CmNAC* genes

The publicly available RNA-seq data from different tissues of chestnut were used to study the expression of *CmNAC* genes and explore their potential functions. Specifically, RNA-seq data of chestnut buds of 20, 25, and 30 days post-anthesis, nuts of 70, 82, and 94 days post-anthesis, and fertile/abortive ovules on 15-July, 20-July, and 25-July were analyzed. The accession numbers of the above RNA-seq data were in Supplementary Table S1, and all of these were three biological replicates. We used the Kallisto software to quantify RNA-seq data into Transcripts Per Kilobase of exon model per Million mapped reads (TPM) (Bray et al., 2016). The “Normalized” function in TBtools was used to normalize gene expression, and we obtained the heat-maps of gene expression based using TBtools.

### qRT-PCR and subcellular location

We collected the nuts of “Yanlong” chestnuts 70, 82, and 94 days after flowering for real-time quantitative PCR experiments, which were stored at −80°C. Specifically, we used the RNAprep pure Plant Kit to extract and isolate RNA, and used the PrimeScript RT Master Mix (Takara, Beijing) to reverse transcribe RNA into single-stranded cDNA. ABI 7500 Real-Time PCR system (Applied Biosystems Inc., Foster City, CA, United States) with TB Green Premix Ex Taq (Takara) was used to conduct the RT-PCR experiments. The specific primer information was shown in Supplementary Table S2, in which the *18S* gene of chestnut was used as the reference gene. The relative gene expression values were calculated using the comparative 2^-△△CT^ method (Livak and Schmittgen, 2001).

## Results

### Identification and physicochemical properties

**FIGURE 1:**
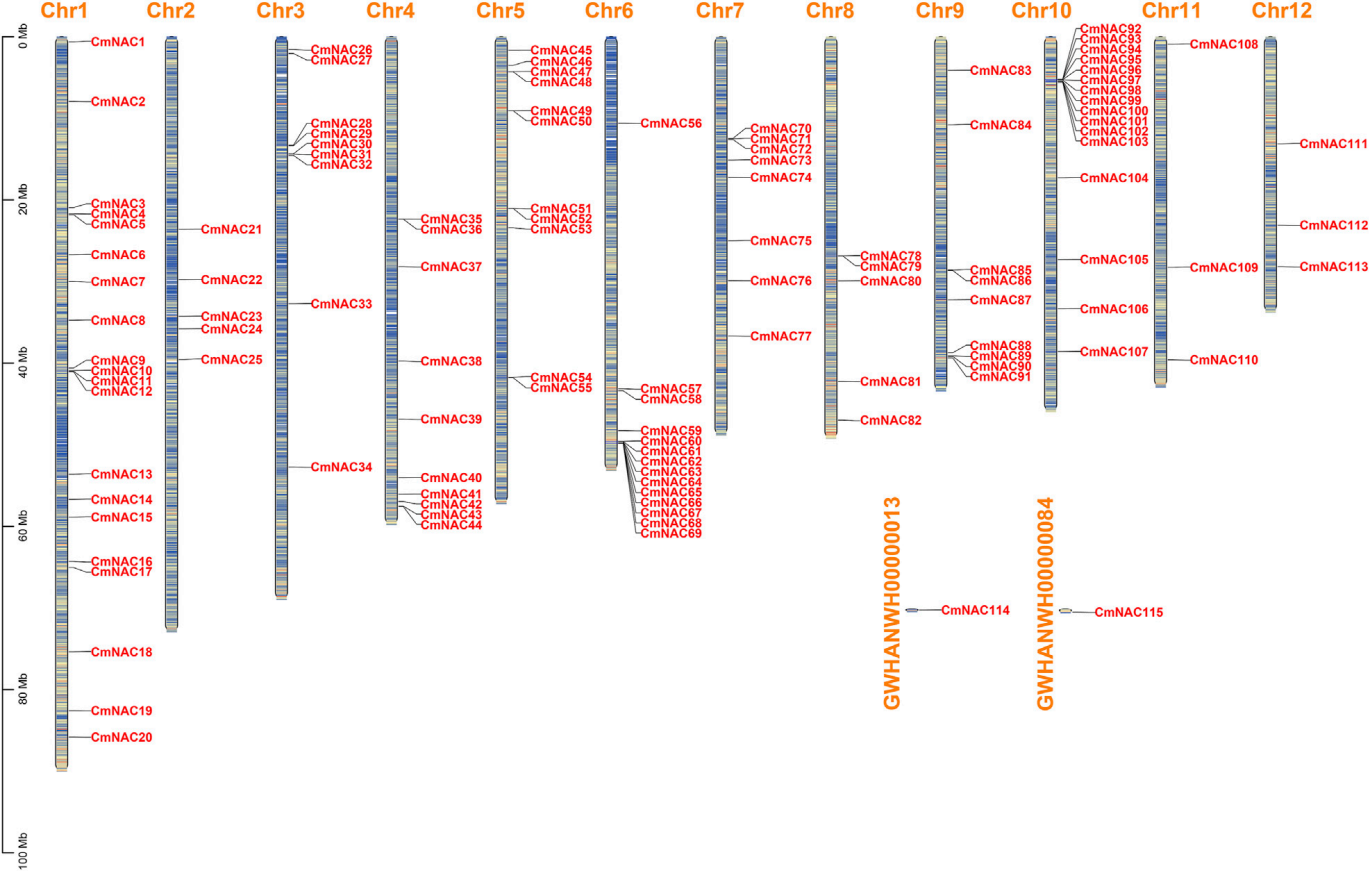
Chromosome distribution of *CmNAC* genes. The color of segments in the chromosomes shows the gene density of the corresponding region.

After strict screening by BLASTP and HMMER 3.0 programs, and using Batch-CDD to ensure the existence of the NAM domain, we finally identified 115 NAC gene family members in the chestnut genome. In addition, 115 *CmNAC* genes were renamed *CmNAC1* to *CmNAC115* based on their relative position. *CmNAC1*∼*CmNAC113* were unevenly distributed on Chr1∼Chr12, while *CmNAC114* and *CmNAC115* were distributed on two scaffolds not attached to chromosomes (Figure 1; Supplementary Table S3). *CmNACs* had the largest number of genes distributed on Chr1 and Chr10, with 20 and 16 members, respectively. *CmNACs* had the least number of genes distributed on Chr11 and Chr12, with only three members. The amino acids number, molecular weight, instability index, and pI of CmNAC gene family members varied greatly (Supplementary Table S3). Specifically, the number of amino acids in CmNACs ranged from 93 (CmNAC64) to 934 aa (CmNAC60), and the molecular weight was between 10,992.77 Da (CmNAC64) and 106,329.77 Da (CmNAC60). The pI values ranged from 4.26 (CmNAC89) to 10.08 (CmNAC64), of which 83 CmNAC proteins were acidic, 31 were basic proteins, and CmNAC45 was a neutral protein. The instability index values of 25 CmNAC proteins were less than 40, and the protein properties were stable, accounting for approximately 21.74% of CmNAC proteins. CmNAC proteins were all hydrophilic proteins, due to the grand average of hydropathicity (GRAVY) values being less than zero. In summary, it is inferred that most of the CmNAC family proteins were unstable alkaline hydrophilic proteins. The subcellular localization predicted that CmNAC proteins were distributed in the nucleus, chloroplast, cytosol, Golgi apparatus, peroxisome, extracellular, mitochondrion, and endoplasmic reticulum, with 69.57% of the proteins located in the nucleus and a few CmNAC proteins located in other organelles (Supplementary Table S3).

### Phylogenetic analysis

**FIGURE 2:**
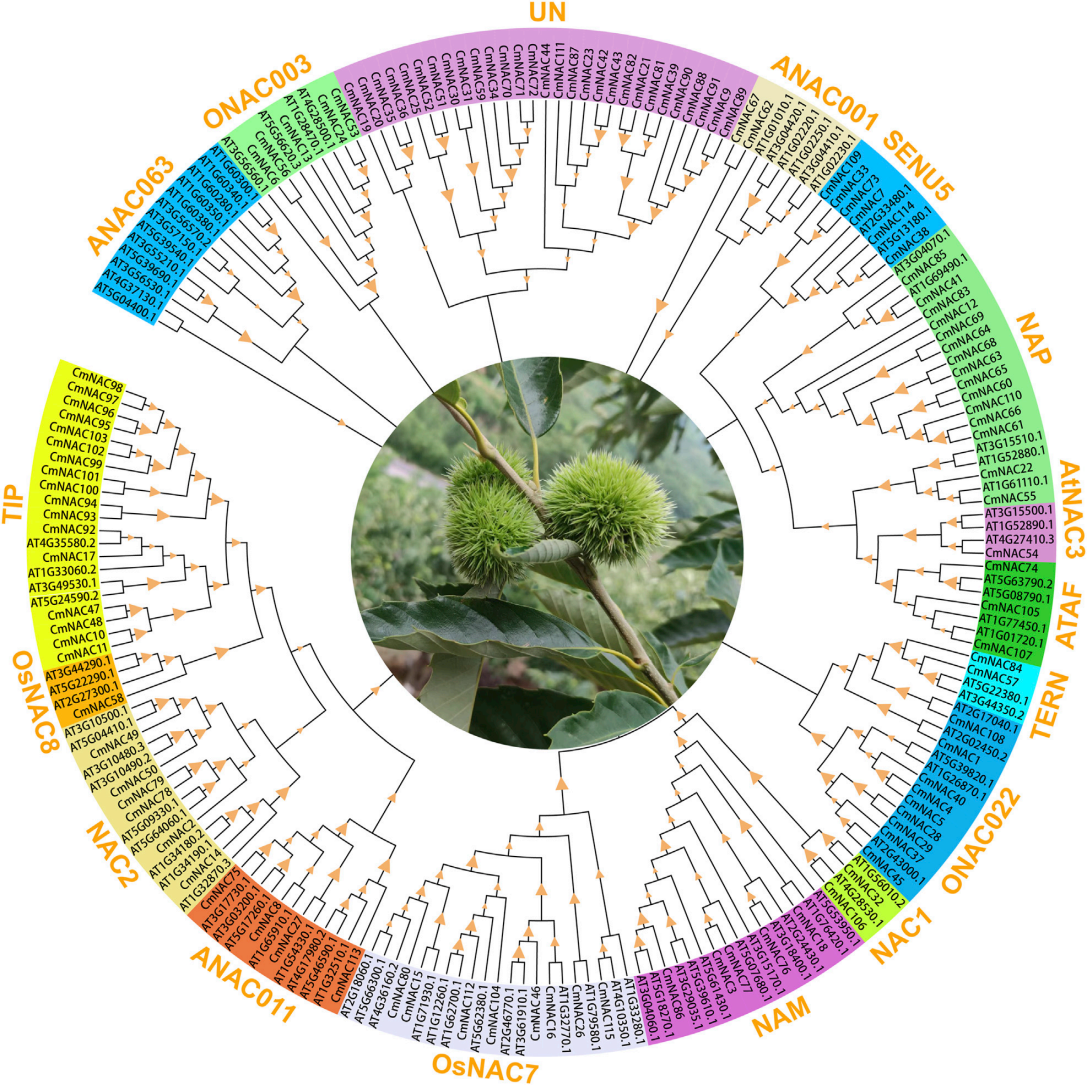
The phylogenetic tree of 209 NAC proteins of chestnut and *A. thaliana*. MEGA 7.0 was used to construct the phylogenetic tree based on the protein sequences with maximum likelihood method. The proteins were clustered into 17 groups. Different background colors indicate the different groups of the NAC proteins.

To clarify the genetic relationships among members of the *CmNAC* genes, a phylogenetic tree with the maximum likelihood method was constructed using a total of 209 NAC protein sequences from chestnut and *A. thaliana* (Figure 2). The results showed that 115 CmNAC proteins were divided into 16 subgroups, namely, ONAC003, ANAC001, SENU5, NAP, AtNAC3, ATAF, TERN, ONAC022, NAC1, NAM, OsNAC7, ANAC011, NAC2, OsNAC8, TIP, and 29 unclassified CmNAC proteins, based on their homology with NAC proteins in *A. thaliana* (Ooka et al., 2004). Interestingly, no CmNAC protein was classified into the ANAC063 subgroup. Except for the UN subgroup, the TIP and NAP subgroups contained the largest number of *CmNAC* genes, with 17 and 15 members, respectively. The OsNAC8 and AtNAC3 subgroups contained the least number of *CmNAC* genes, both with only one member. Furthermore, we also constructed phylogenetic trees of *NAC* genes in chestnut and *A. thaliana* with the neighbor-joining method (Supplementary Figure S1). These results indicated that CmNAC proteins exhibit a certain degree of diversity, which was similar to the phylogenetic results of NAC proteins reported in many species (Jin et al., 2020; Jia et al., 2021; Du et al., 2022).

### Gene structure, motif, and *cis*-acting elements

**FIGURE 3:**
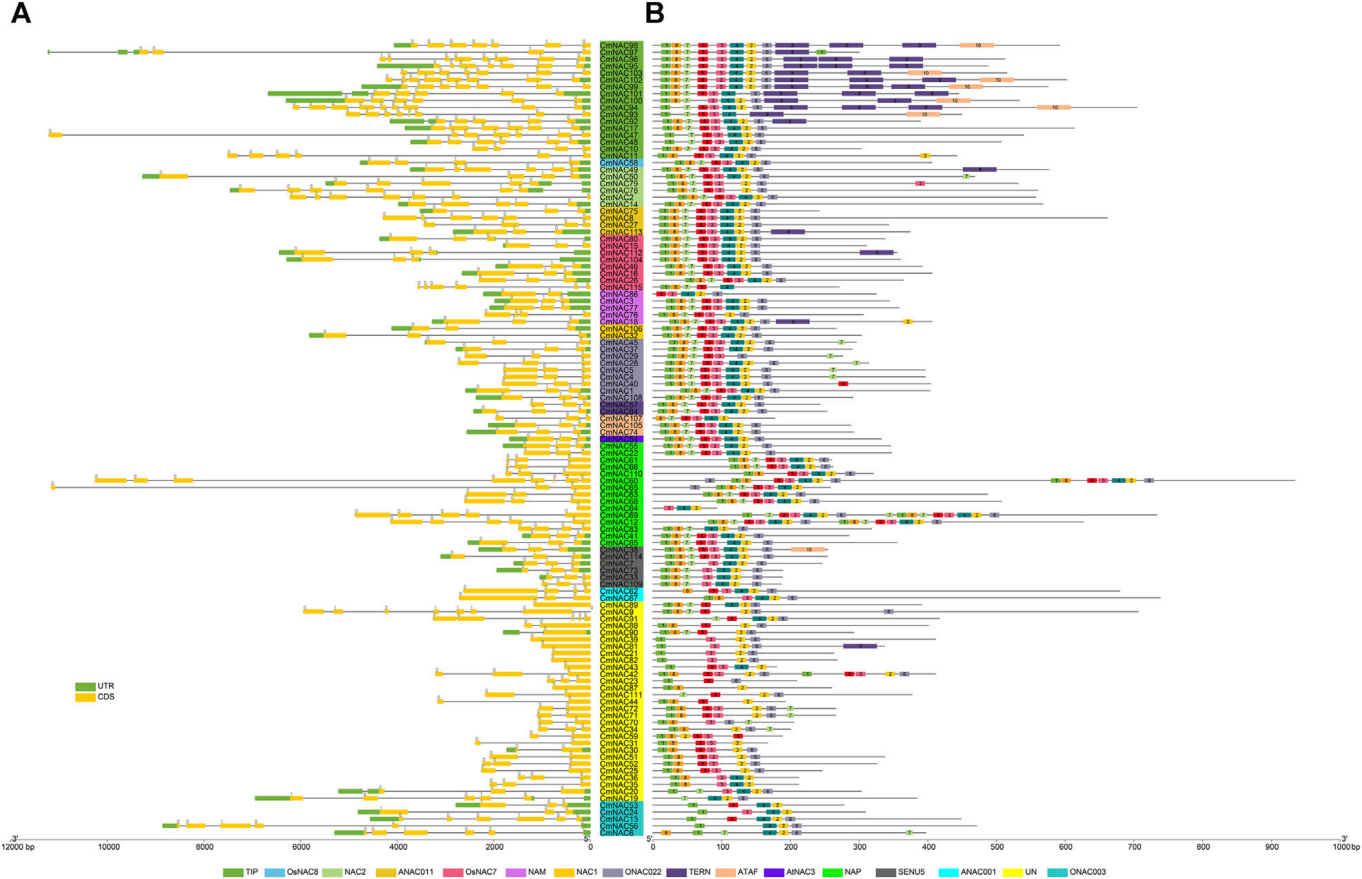
The gene structure and conserved motifs distribution of *CmNAC* genes. **(A)** Gene structure of *CmNAC* genes. **(B)** Distribution of conserved motifs of *CmNAC* genes. The gene names highlighted in different colors in the middle are classified according to the *CmNACs* obtained from the phylogenetic tree.

We analyzed the gene structure, motif distribution, and promoter *cis*-elements to further understand *CmNAC* genes. The phylogenetic tree constructed using only 115 CmNAC protein sequences was consistent with the phylogenetic tree constructed using 209 NAC protein sequences from chestnut and *A. thaliana* (Figure 2; Supplementary Table S1–3). Specifically, Gene Structure Display Server 2.0 (GSDS) and MEME (http://meme.nbcr.net/meme/intro.html) were used to analyze the distribution of exons and introns in the *CmNAC* genes, and conserved motifs (Figure 3). The structure analysis of the *CmNAC* genes showed a high degree of difference in the number of introns and exons, with approximately 1–10 introns present, and the gene structure of *CmNAC* transcription factors in the same subfamily was relatively conserved. In addition, the introns of most *CmNAC* genes were phase zero, and this further indicated that they were conserved in gene structure. Diversified motif compositions were detected in the protein sequence of the *CmNAC* genes. Interestingly, some motifs can be detected in almost all *CmNACs*, which may be related to the shared domains of the family. In addition, *CmNAC* members within the same subgroup had a relatively consistent motif distribution. For example, members in the NAC1 subgroup had a completely consistent motif distribution, while motif 9 mainly existed in the TIP subgroup. Some subgroups had individual members missing motifs that other members all contained. For example, the *CmNAC115* in the OsNAC7 subgroup lacked motif 3, which was preserved in other members of the OsNAC7 subgroup (Figure 3B). Indeed, the specific functions of these motifs required further in-depth research (Yang et al., 2023).

**FIGURE 4:**
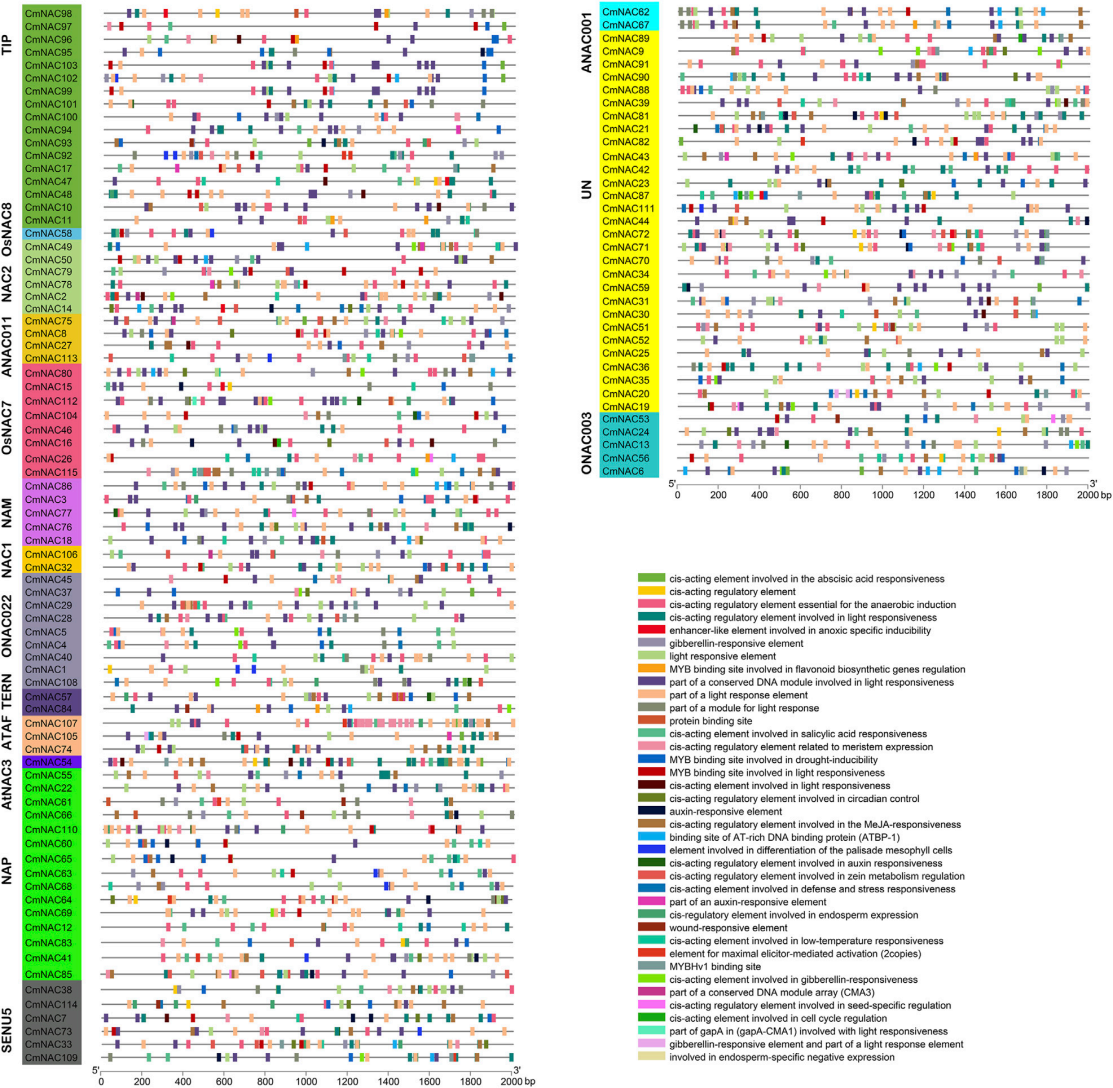
*Cis*-acting elements in the promoter region of *CmNAC* genes. The gene names highlighted in different colors on the left are classified based on *CmNAC* obtained from the phylogenetic tree. The distribution of *cis*-acting elements in the 2,000 bp upstream promoter is shown. The different functions of *cis*-acting elements are represented by different colors, as shown on the right.

The *cis*-acting elements analysis was necessary to understand the functions of *CmNAC* genes. A total of 2,887 *cis*-acting elements were identified by predicting the upstream 2000 bp sequences of 115 *CmNAC* gene promoters, and these *cis*-acting elements involve various types of functions (Figure 4; Supplementary Table S4). For example, TGA-element or TGA-box related to auxin response was identified in the promoter regions of 31 *CmNAC* genes. Gibberellin-responsive elements were identified in the promoter regions of 56 *CmNAC* genes, and *cis*-regulatory elements involved in endosperm expression were identified in the promoter regions of 20 *CmNAC* genes. These results suggested that members of the *CmNAC* gene family were closely related to the growth and development of chestnut. Additionally, a large number of *cis*-acting elements related to plant resistance to stress were identified, such as wound-responsive elements, *cis*-acting elements involved in defense and stress responsiveness (Figure 4; Supplementary Table S4) (Sun et al., 2022).

### Gene duplication and collinear analysis of *CmNAC* genes

**FIGURE 5:**
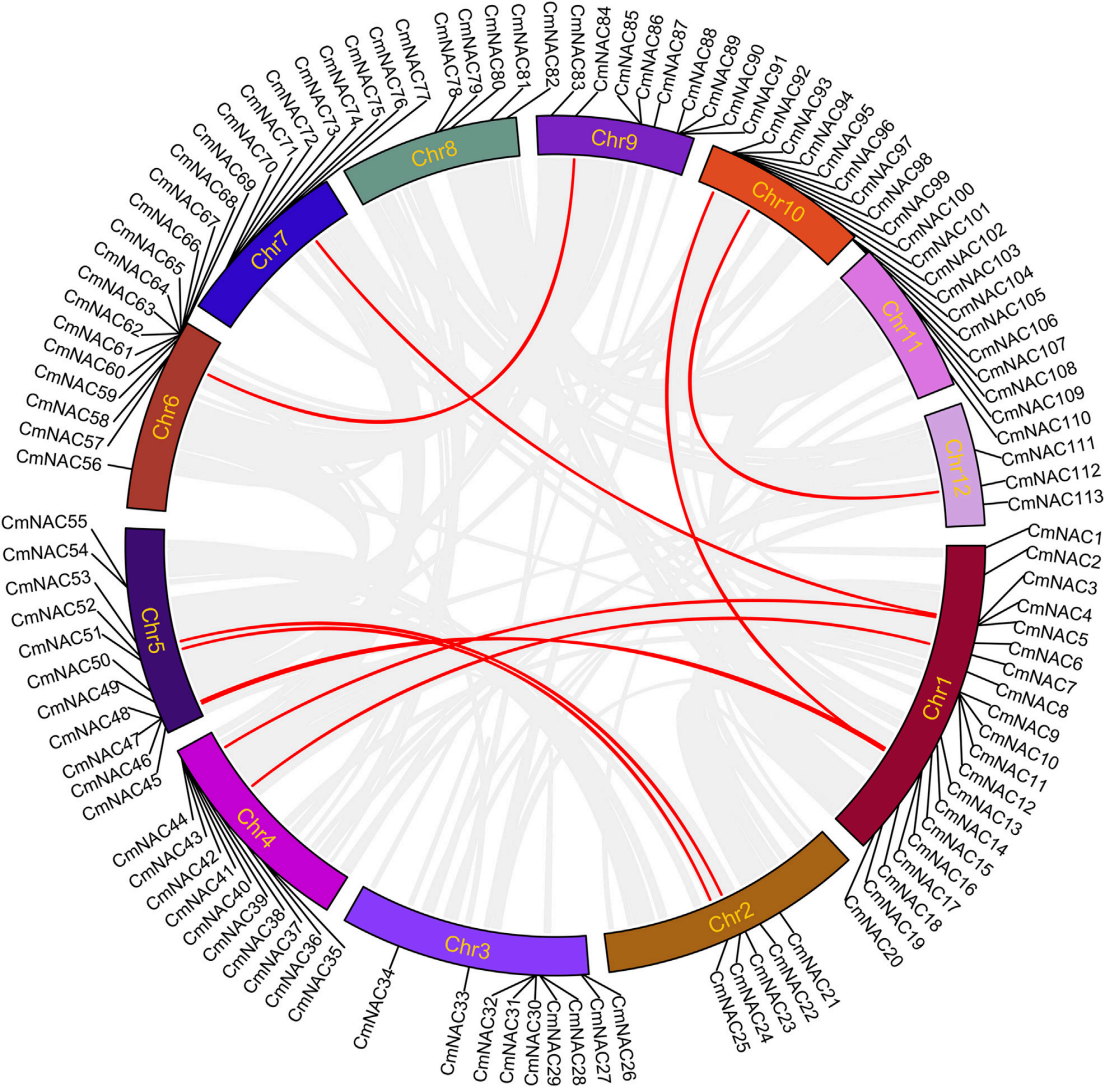
Circos plot showing the collinearity of the *CmNAC* genes. The homologous gene pairs formed by the chestnut *NAC* genes in the collinear region are connected by red lines, while other collinear regions are connected by light gray lines.

**FIGURE 6:**
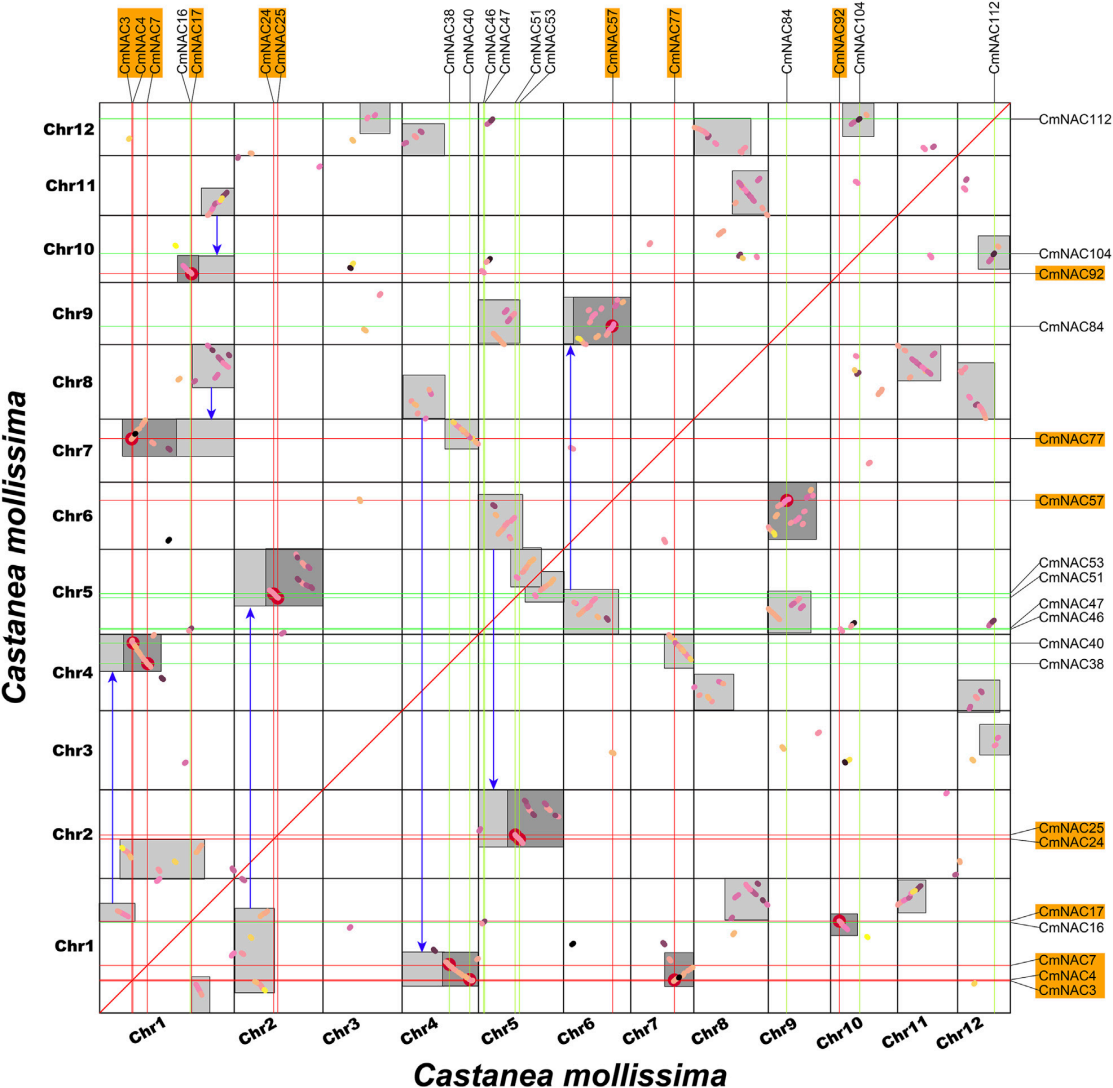
Homologous collinearity dot-plot within the chestnut genome. The collinear blocks from WGD containing *CmNAC* genes are marked in the orange boxes of the figure. The boxes in the figure represent collinear regions within the chestnut genome, in which the dark or light highlighted boxes indicate regions formed by WGD event containing *CmNAC* homologous gene pairs and complementary fragments forming larger homologous regions, respectively.

Gene duplication is one of the reasons for the formation of gene families and the diversification of gene functions (Liu and Widmer, 2014; Quan et al., 2019; Yu et al., 2023a). We conducted collinear analysis on the chestnut genome using MCScanX to explore duplication events among members of the CmNAC gene family (Figure 5). Fifty-three dispersed duplicate genes were found among 115 *CmNAC* genes (Supplementary Table S5). In addition, five and four *CmNAC* genes were identified to be proximal and tandem duplications, respectively. Interestingly, 32 of the *CmNAC* genes were considered WGD or segmental duplication. A homologous dot-plot of the chestnut genome and color distinguished them based on the Ks value of homologous genes was drawn to preliminarily determine homologous genes from different duplication events (Figure 6). Furthermore, referring to our previous distribution of Ks values of WGD of the chestnut genome and the complementary relationship between homologous gene fragments (Yu et al., 2023b), we ultimately confirmed 9 pairs of 18 *CmNAC* genes from the chestnut WGD event. The final results indicated that dispersed duplication and WGD were the main drivers of *CmNAC* gene expansion (Supplementary Table S5).

**FIGURE 7:**
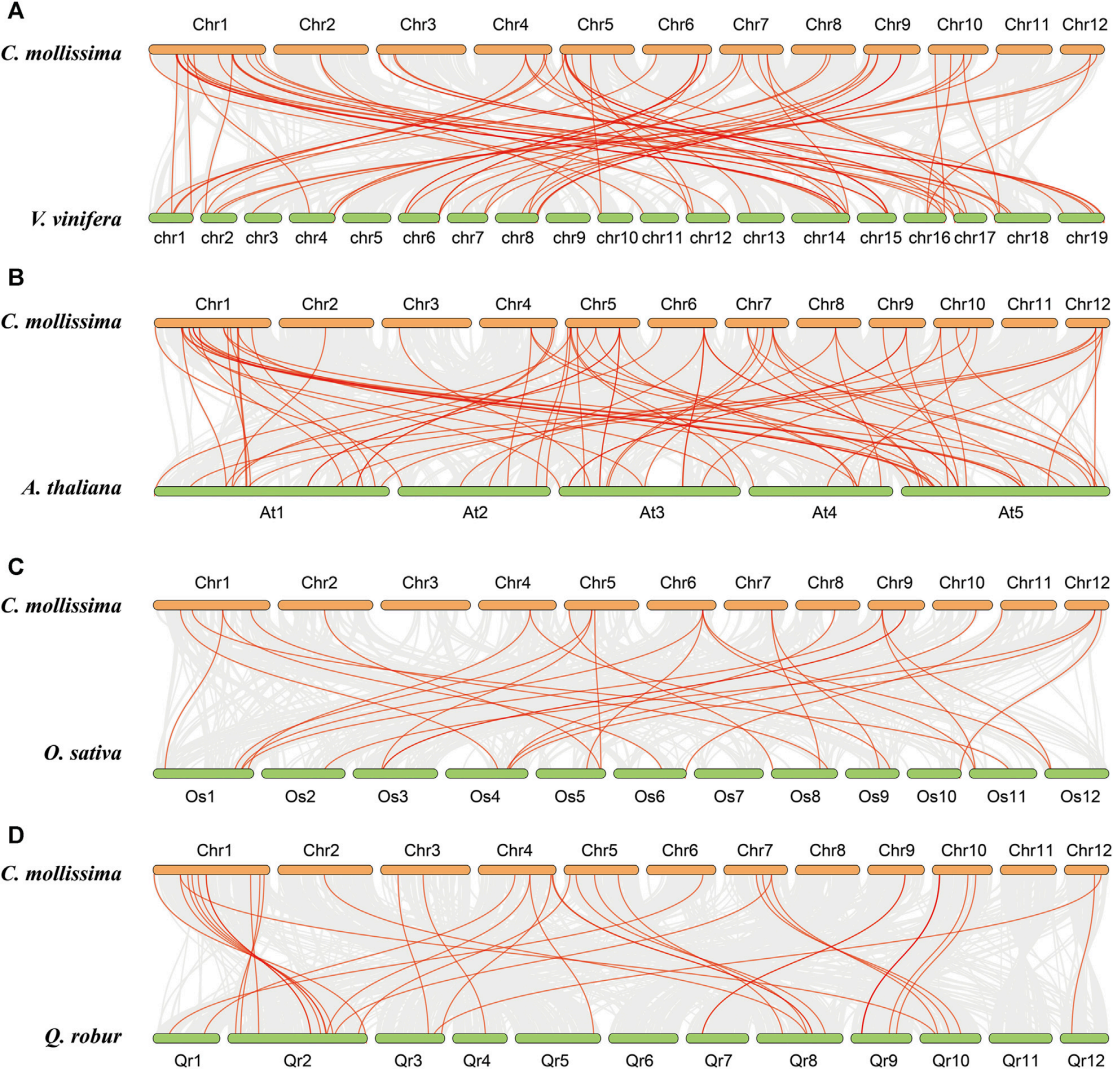
Collinear relationships with chestnut, grape, *A. thaliana*, rice and oak genomes. **(A)** Collinear relationship between chestnut and grape genomes. **(B)** Collinear relationship between chestnut and **(A)** *thaliana* genomes. **(C)** Collinear relationship between chestnut and rice genomes. **(D)** Collinear relationship between chestnut and oak genomes.

The collinear relationships between chestnut and four representative species, namely, *A. thaliana*, rice, grape, and oak, were analyzed to further understand the evolution of the *CmNAC* genes (Figure 7). The results showed that 65 collinear blocks were found between the genomes of chestnut and grape, with an average block length of 49.23 (Supplementary Table S6). The number of collinear blocks found between the genomes of chestnut and *A. thaliana*, rice, and oak was 66, 28, and 38, respectively, with average block lengths of 21.08, 10.61, and 26.29, respectively (Supplementary Table S7–9). Furthermore, we have discovered 71 orthologous gene pairs containing a total of 50 *CmNAC* genes between the genomes of chestnut and grape (SSupplementary Table S10). However, only 42 (73 pairs), 19 (29 pairs), and 36 (39 pairs) *CmNAC* genes were found between chestnut and *A. thaliana*, rice, and oak genomes, respectively (Supplementary Table S11–13). These results indicated that better collinearity was preserved between the genomes of chestnut and grape. In addition, we calculated the Ka/Ks values of gene pairs formed by different duplication models (such as tandem, WGD, and segmental duplication), which suggested that they experienced purification selection pressure during their evolution process (Ka/Ks < 1) (Supplementary Table S14) (Anisimova et al., 2001; Roth and Liberles, 2006).

### Expression analysis of *CmNAC* genes

**FIGURE 8:**
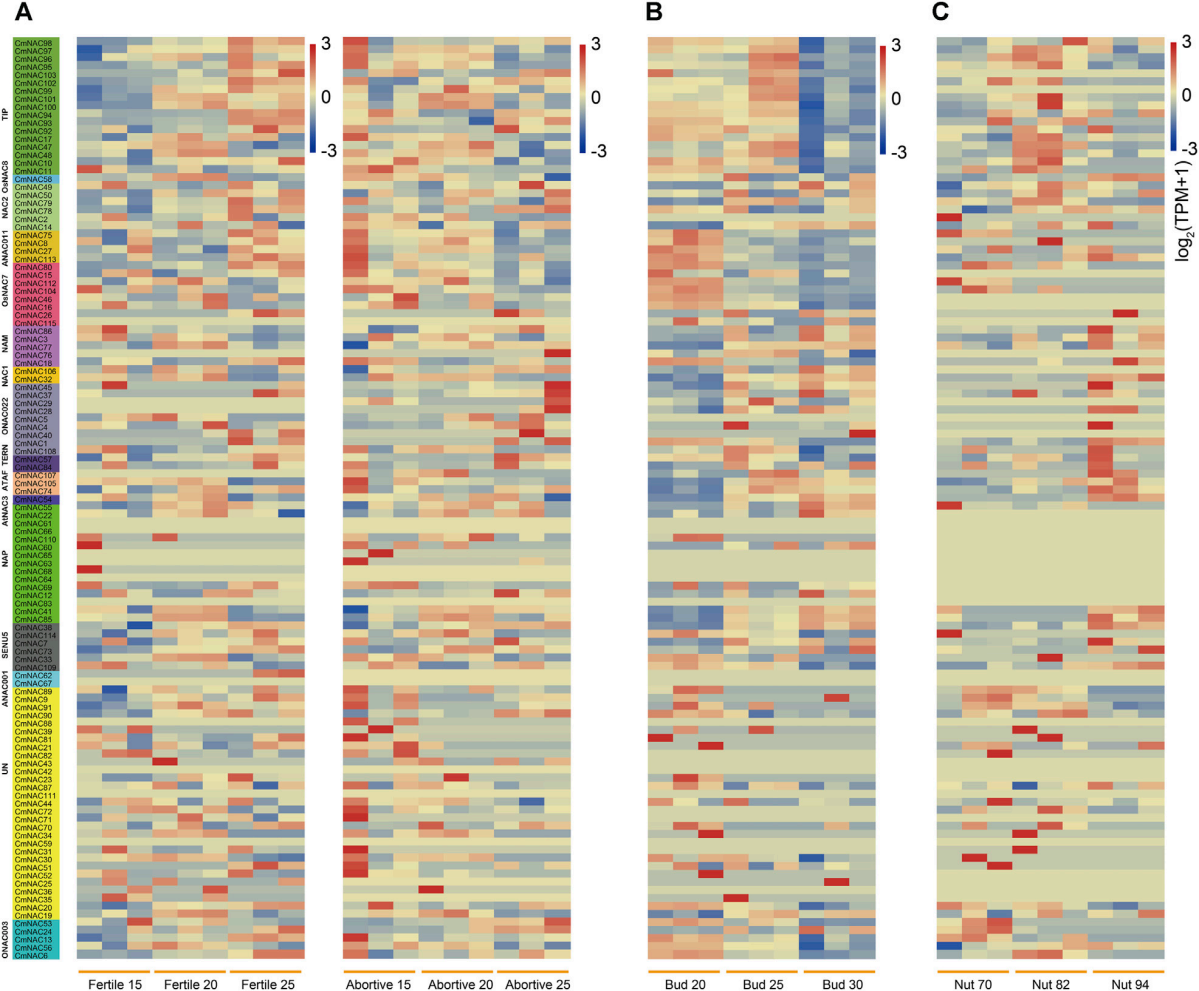
Heat-map of *NAC* genes expression in ovules (fertile/abortive), buds and nuts of chestnut at different stages. **(A)** Heat-map of *NAC* genes expression in fertile and abortive ovules of chestnut at 15-July, 20-July, and 25-July. The gene names highlighted in different colors on the left are classified based on *CmNAC* obtained from the phylogenetic tree. **(B)** Heat-map of *NAC* genes expression in chestnut buds 20, 25, and 30 days after flowering. **(C)** Heat-map of *NAC* genes expression in chestnut nuts 70, 82, and 94 days after flowering.

To further understand the possible functions of *NAC* genes in different tissue development processes of chestnuts, we fully utilized RNA-seq from the NCBI database of chestnut ovules (fertile/abortive), buds, and nuts (Figure 8). Some *CmNAC* genes exhibited high expression levels throughout the entire developmental stage of fertile ovules, while they were almost not expressed throughout the entire developmental process of abortive ovules (Figure 8A). For example, the TPM values of *CmNAC113* in the three developmental stages of fertile ovules were 20.71, 13.17, and 44.92, respectively, while the TPM values were 5.82, 1.70, and 2.15 in the same stage of abortive ovules, respectively. On the contrary, some *CmNAC* genes were almost not expressed throughout the entire developmental stage of fertile ovules, but exhibited higher expression levels in abortive ovules. For example, the TPM values of *CmNAC109* in the three developmental stages of fertile ovules were 1.149, 0.67, and 0.19, respectively, while the TPM values were 26.74, 7.12, and 25.76 in the same stage of abortive ovules, respectively. Notably, some *cis*-regulatory elements related to endosperm expression were found in the promoter regions of these *CmNAC* genes (Figure 4). These results indicated that these *NAC* genes in chestnut were related to the fertility of chestnut endosperm.

**FIGURE 9:**
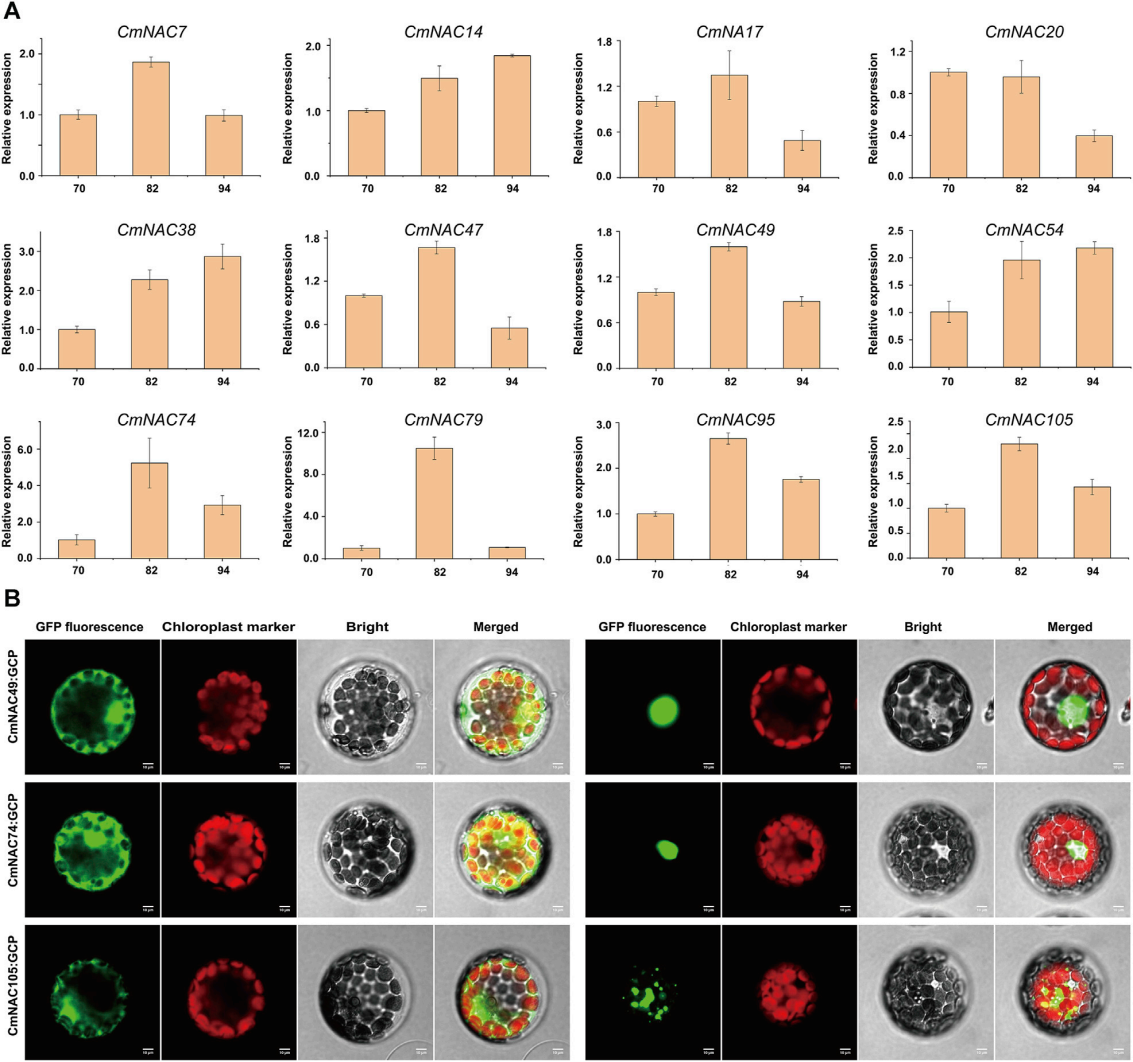
qRT-PCR of 12 *CmNAC* genes in chestnut nuts at different developmental stages and subcellular location of three *CmNAC* genes proteins in **(A)** *thaliana* protoplasts. **(A)** qRT-PCR of *CmNAC7*, *CmNAC14*, *CmNAC17*, *CmNAC20*, *CmNAC38*, *CmNAC47*, *CmNAC49*, *CmNAC54*, *CmNAC74*, *CmNAC79*, *CmNAC95* and *CmNAC105* in chestnut nuts at different developmental stages. **(B)** Subcellular location of *CmNAC49*, *CmNAC74* and *CmNAC105* proteins in **(A)** *thaliana* protoplasts.

Furthermore, we analyzed the expression levels of *CmNAC* genes in chestnut buds and nuts at different developmental stages (Figure 8B). Interestingly, we found that some *CmNAC* genes maintained high expression levels, and their expression levels continued to increase, during chestnut bud development. For example, the expression level of *CmNAC14* in chestnut buds 20 days after flowering was 66.12 TPM, while the expression levels in chestnut buds 25 and 30 days after flowering were 126.49 and 148.57 TPM, respectively. The expression level of *CmNAC38* in chestnut buds 20 days after flowering was 90.05 TPM, while the expression levels in chestnut buds 25 and 30 days after flowering were 427.51 and 871.72 TPM, respectively. Meanwhile, gibberellin-responsive element and *cis*-acting regulatory element related to meristem expression were identified in the promoter regions of *CmNAC14* and *CmNAC38* (Figure 4). Notably, gibberellin is a very important plant hormone that participates in many biological processes, such as the development and differentiation of plant buds (Richards et al., 2001). In addition, during the development of chestnut nuts, some *CmNAC* genes remained highly expressed, such as the TPM values of *CmNAC49* at 70, 82, and 94 days after flowering of chestnut nuts, which were 122.94, 166.93, and 123.73, respectively (Figure 8C). Some *CmNAC* genes exhibited sharp changes in expression with the development of chestnut nuts. For example, *CmNAC54* was almost not expressed in nuts at 70 and 82 days after flowering (TPM values of 0.17 and 0.19, respectively), while the expression level increased sharply at 94 days after flowering (chestnut maturity), reaching a TPM value of 72.39. Interestingly, *cis*-acting elements involved in abscisic acid and MeJA responsiveness were identified in *CmNAC49* and *CmNAC54* (Figure 4). The expression patterns of 12 *CmNAC* genes that we were interested in during the development of chestnut nuts were validated using qRT-PCR experiments (Figure 9A). These results showed that the qRT-PCR experiment results and RNA-seq analysis showed consistent expression patterns of *CmNAC* genes. For example, the expression level of *CmNAC38* continuously increased until it reached its highest level at maturity (94 days after flowering). *CmNAC47* has the lowest expression level on 70 days after flowering, the highest expression level on 82 days after flowering, and a significant decrease in expression level at maturity (94 days after flowering). These results suggested that some *CmNAC* genes may play important roles in the development of chestnut buds and nuts. Additionally, to determine the subcellular localization of *CmNAC* genes through experiments, we randomly selected three *CmNAC* members, namely, *CmNAC49*, *CmNAC74*, and *CmNAC105*, and instantaneously expressed GFP-CmNAC49, GFP-CmNAC74, and GFP-CmNAC105 fusion proteins in *A. thaliana* protoplasts for subcellular localization (Figure 9B). Confocal microscopy analysis showed that the GFP-fused *CmNAC49* and *CmNAC74* displayed fluorescence signals distributed in the nucleus, and GFP-fused *CmNAC105* was localized to the nucleus and peroxisome. These results were consistent with the subcellular localization prediction of *CmNAC* through bioinformatics analysis.

## Discussion

The NAC TF family is one of the largest transcription regulatory factor families in plants (Du et al., 2022). However, the NAC gene family in many plants has been identified, but there have been no relevant reports on the *NAC* genes in chestnut. Here, 115 non-redundant *NACs* were identified in the chestnut genome, similar to the number of *NACs* in pepper (*Capsicum annuum*) (104) (Diao et al., 2018) and *Dendrobium officinale* (110) (Yang et al., 2023), but less than that in cabbage (*Brassica rapa*) (188) (Ma et al., 2014), *Malus pumila* (180) (Su et al., 2013), sunflower (*Helianthus annuus*) (150) (Li et al., 2021) and rice (151) (Nuruzzaman et al., 2010). The difference in size may be due to differences in WGD and other gene duplication events that accompany species evolution and differentiation. The analysis of physicochemical properties showed that 115 CmNAC proteins showed significant differences in amino acid length, relative molecular weight, and pI, but were relatively conserved in gene structure. The vast majority of *CmNAC* genes had 1–10 introns, and similar results were found in *NAC* genes in species such as *Coffea canephora* (Dong et al., 2019) and *Nicotiana tabacum* (Li et al., 2018). CmNAC proteins had high motif conservation, and the motif composition of CmNAC proteins in the same subfamily was basically similar, but there were also a few CmNAC protein motifs that were clustered together and had different compositions. For example, *CmNAC115* in the OsNAC7 subgroup lacked motif 3 that other members of the subgroup had, which may be due to the loss of motif 3 in *CmNAC115* during evolution, while other shared sequences of the subgroup were preserved.

In this study, maximum likelihood and neighbor-joining methods were used to construct phylogenetic evolution trees of the NAC gene family members in chestnut and *A*. *thaliana*, respectively, which were divided into 17 subgroups (Figure 2; Supplementary Figure S1). The results of the inter species phylogenetic tree constructed separately by the NAC family of chestnut and jointly by chestnut and *A*. *thaliana* were consistent (Supplementary Figure S1–3). Similar protein sequences usually imply similar functions (Yu et al., 2023b). Previously, it was reported that *ANAC031* (*AT1G76420*) and *ANAC098* (*AT5G53950*) in *A*. *thaliana* played important roles in meristem formation and organ boundary establishment (Spinelli et al., 2011). Due to the close homologous relationship between *CmNAC18* and *ANAC031*, *ANAC098*, and, it is speculated that *CmNAC18* may be related to the development of chestnut buds or roots. Notably, RNA-seq data analysis found that the expression level of *CmNAC18* during the early stage of chestnut bud development (20 days after flowering) was eight times higher than that at 25 days after flowering, and it was almost not expressed at 30 days after flowering (with an average TPM of 0.16) (Figure 8B). In addition, the gibberellin-responsive element was identified in the promoter region of *CmNAC18* (Figure 4) and gibberellin has been proven to be associated with bud development in multiple species (Richards et al., 2001; Zhang et al., 2020). These results further supported the involvement of *CmNAC18* in the development of chestnut buds. Similarly, *ANAC054* (*AT3G15170*) and *ANAC098* are the key genes that regulate the development of flower organs, affecting the development of stamens and pistils (Krizek and Fletcher, 2005). Their functional deficiency may lead to abnormal embryonic development. In addition, *ANAC054* and *ANAC098* can promote the formation of carpel margin meristem (CMM) (Kamiuchi et al., 2014), but there are defects in the development of septum and ovule in the *A*. *thaliana ANAC054*/*ANAC098* double mutant (Ishida et al., 2000; Cucinotta et al., 2018). *CmNAC77* and *CmNAC86* belonged to the same NAM subgroup as *ANAC054* and *ANAC098*, and had good sequence similarity with them (Figure 2). In addition, the expressions of *CmNAC77* in fertile ovules (TPM values for three developmental stages: 20.47, 47.13, and 23.78) were significantly lower than that in abortive ovules (TPM values for three developmental stages: 145.63, 303.67, and 288.45), while the expression of *CmNAC86* in fertile ovules (TPM values for three developmental stages: 4.22, 1.81, and 1.38) was significantly lower than that in abortive ovules (TPM values for three developmental stages: 8.13, 12.56 and 15.88) (Du et al., 2021). These results suggested that *CmNAC77* and *CmNAC86* may have important potential roles in the fertility of chestnut ovules.

There is ample evidence to suggest that the NAC gene family already existed in early plants such as moss and ferns, and its origin is closely related to the formation of the multicellular structure and complex life cycle of plants (Hu et al., 2022; Yang et al., 2023). The expansion of the NAC gene family is mainly achieved through gene duplication and selective preservation (Diao et al., 2018; Jin et al., 2020). Gene duplication includes WGD, segmental, proximal, dispersed, and tandem duplication (Wang et al., 2012). WGD events have occurred multiple times in the evolutionary history of many plants, leading to the large-scale expansion of gene families (Wang et al., 2012). For example, WGD was an important driving force for the evolution and expansion of the plant PINOID gene family across plant species (Bai et al., 2022). Two additional WGDs after the core eudicot common hexaploidization (ECH) event increased the number of members in the GRAS and BBX gene families in sea buckthorn (*Hippophae rhamnoides*), exceeding the corresponding gene family members in grapes that did not experience additional WGD events and rice that only experienced one WGD event after the ECH event (Yu et al., 2021; Yu et al., 2022; Yu et al., 2023c). In this study, 115 *CmNAC* genes were identified in chestnut, with fewer than in cabbage (188) (Ma et al., 2014), *Malus pumila* (180) (Su et al., 2013), and sunflower (150) (Li et al., 2021), which all experienced multiple WGD events (Badouin et al., 2017; Walden et al., 2020; Su et al., 2021). The relatively small number of *NAC* genes in the chestnut genome may be due to its lack of additional WGD events after the ECH event (Yu et al., 2022). In addition, the lack of additional WGD events in both chestnut and grape genomes may be the reason for better collinearity between them.

Here, 115 *NACs* were identified and systematically characterized in the chestnut genome, and the expression profiles at different developmental stages of chestnut buds, nuts, and ovules (fertile/abortive) were analyzed. These *CmNACs* were classified into 16 subgroups and phylogenetic trees were constructed based on *NAC* genes in chestnut and *A*. *thaliana*. The results of the collinear analysis indicated that dispersed duplication had the greatest contribution to the expansion of the NAC gene family in chestnut. Based on RNA-seq data analysis of different tissues in chestnuts at different stages, some *CmNAC* members that may be related to chestnut bud and nut development, as well as ovule fertility were screened. This series of studies provided systematic information about the *CmNAC* genes and will promote further research on its potential function in the future.

## Data Availability

The original contributions presented in the study are included in the article/Supplementary Material, further inquiries can be directed to the corresponding author.
