# Supplementary figures and images for: Genome-wide identification, evolution, and expression analysis of the NAC gene family in chestnut (Castanea mollissima)
Source: Front Genet. 2024 Jan 25;15:1337578. doi: 10.3389/fgene.2024.1337578 (PMC10850246; doi:10.3389/fgene.2024.1337578)

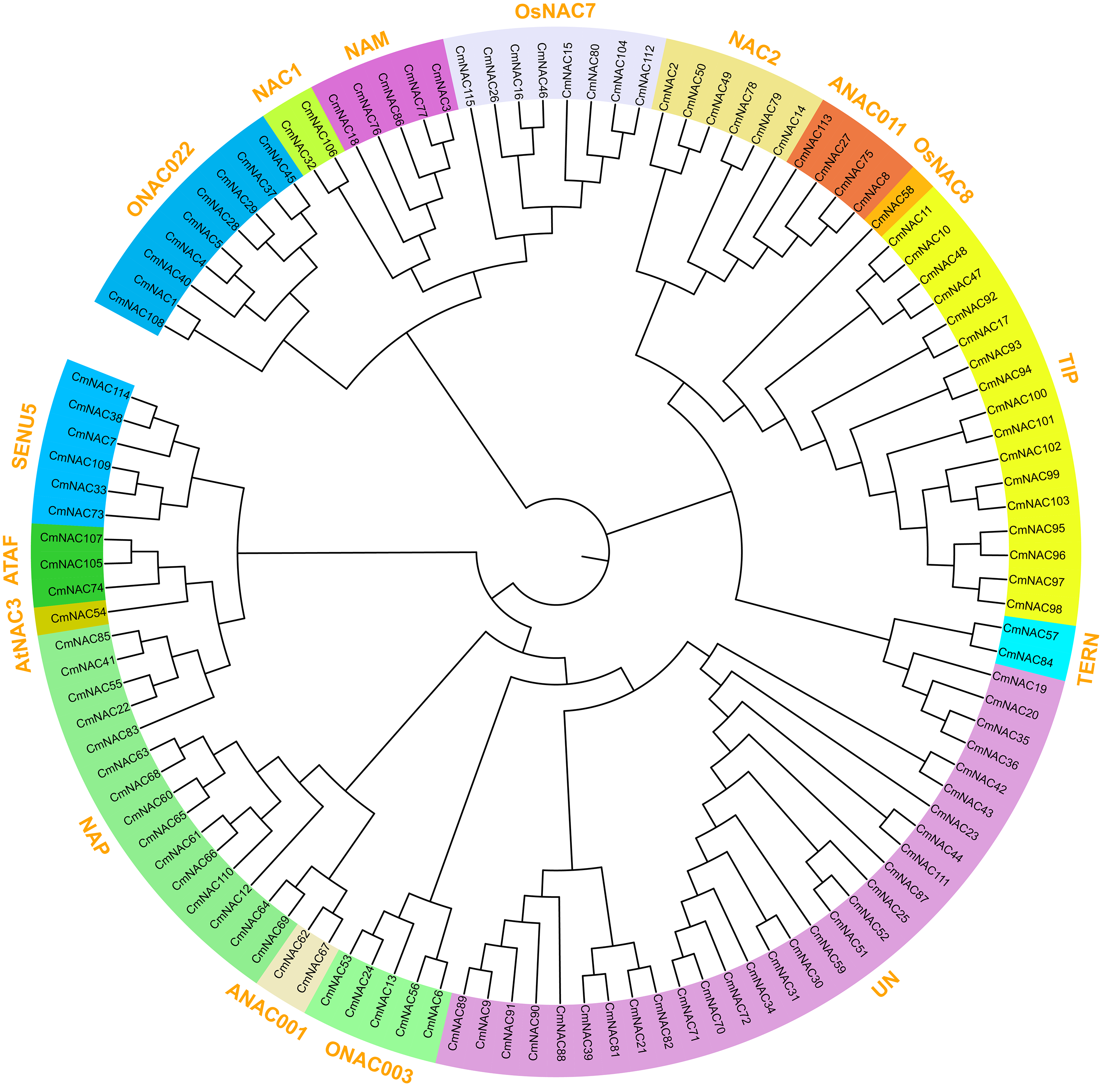

Supplement: Supplementary file 1 [file Image3.TIF]

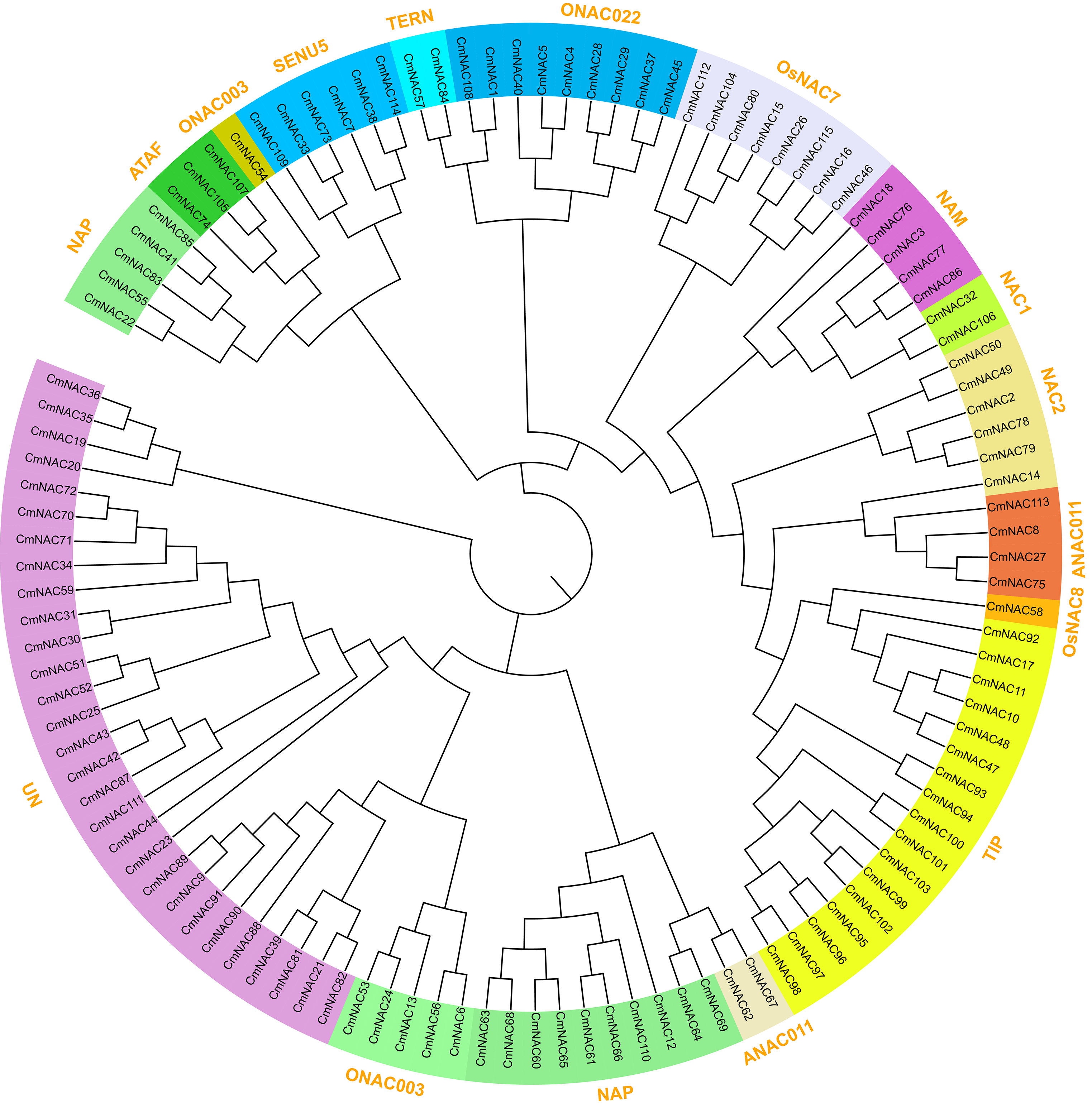

Supplement: Supplementary file 2 [file Image2.TIF]

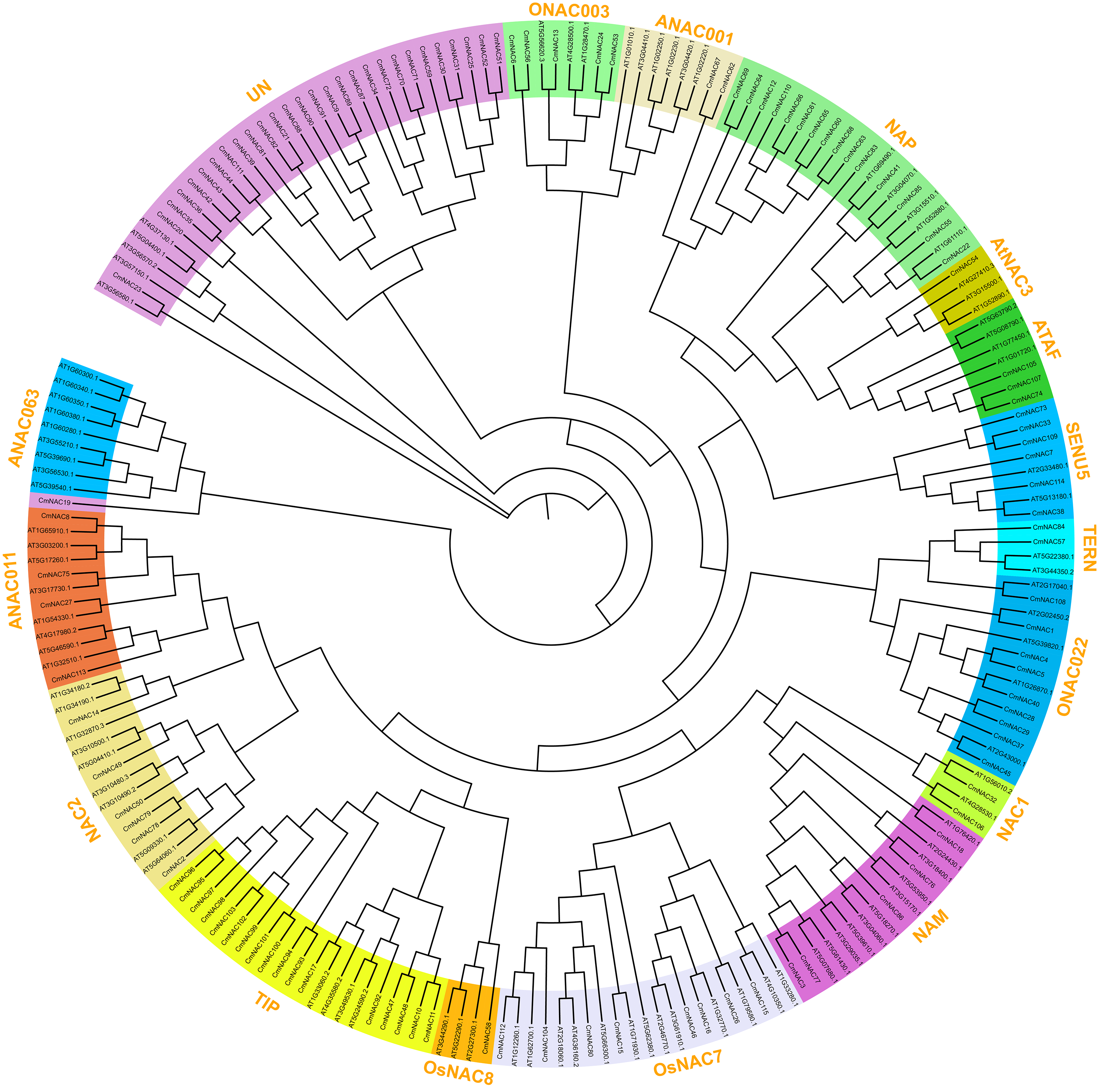

Supplement: Supplementary file 3 [file Image1.TIF]
